# Comparative Crystal Properties of Fluorite and Dolomite: Implications for Flotation Reagent Design

**DOI:** 10.3390/molecules30183770

**Published:** 2025-09-17

**Authors:** Weiwei Wang, Zhengyao Li, Zhenyu Wang, Shaochun Hou, Zhengyuan Zhou, Chunlei Guo, Weiyao Zhu

**Affiliations:** 1 School of Resources and Security Engineering, University of Science and Technology Beijing, Beijing 100083, China; viviw91@163.com (W.W.);; 2 State Key Laboratory of Baiyun Obo Rare Earth Resource Researches and Comprehensive Utilization, Baotou Research Institute of Rare Earths, Baotou 014030, China; 3 State Key Laboratory of High-Efficient Mining and Safety of Metal Mines of Ministry of Education, University of Science and Technology Beijing, Beijing 100083, China

**Keywords:** fluorite, dolomite, crystal properties, cleavage characteristics, density functional theory

## Abstract

This study systematically compares the crystal properties of fluorite (CaF_2_) and dolomite [CaMg(CO_3_)_2_] through first-principle calculations. Density functional theory (DFT) simulations revealed fundamental differences in structural and electronic characteristics: fluorite exhibits purely ionic Ca-F bonds (Mulliken population: 0.08) with a wide bandgap; whereas dolomite demonstrates a hybrid bonding nature featuring ionic Ca-O/Mg-O bonds (populations: 0.09/0.18) and covalent C-O bonds (0.86), which are accompanied by a narrower bandgap. The charge density and density of states (DOS) analyses demonstrated fluorine’s dominant electronic reactivity in fluorite (F 2p states near Fermi level) versus the oxygen/calcium activity in dolomite. Cleavage studies identify preferential fracture planes, with fluorite’s {111} plane exhibiting higher unsaturated bond density (14.76 nm^−1^) than dolomite’s {104} plane (10.55 nm^−1^), which correlates with their distinct mechanical processing behaviors. This work establishes a theoretical foundation for developing selective separation strategies by exploiting crystal-specific surface properties.

## 1. Introduction

Fluorite is one of the most important fluorine-bearing minerals, and it is widely used in chemistry, metallurgy, cement production, ceramics, and various other industries [1,2,3,4,5]. Furthermore, fluorite resources occupy an extremely crucial position in the development of a powerful nation [6,7]. Driven by rapid industrial and economic growth, fluorite resources are consumed in large quantities, necessitating the processing of complex and refractory ores as an inexorable trend. However, exploiting these ores entails more complex processes, leading to significant environmental and economic challenges. Therefore, research on utilizing complex fluorite resources must be intensified.

Carbonate-fluorite ore is a major type of fluorite deposit, as fluorite typically coexists with gangue minerals, such as dolomite and calcite [8,9,10]. The efficient separation of fluorite from dolomite presents significant challenges due to their extremely similar floatability. Research into the structural and electronic properties of these minerals is crucial for elucidating the flotation mechanism of fluorite. Investigations of the electronic properties provided valuable insights into the mineralogical characteristics during flotation, while the atomic-level property analyses of both crystal structures were essential for developing targeted separation strategies.

The crystal structure significantly affects mineral surface activity [11]. First-principle computational studies of crystal structures are a mature and important means for investigating crystal properties. Frontier orbital theory has also been widely used to study mineral–flotation reagent interactions. Research on the electronic structures of Ti_3_O_5_ and Al_2_TiO_5_ anorthite crystals combined with flotation experiments has demonstrated that differing crystal properties lead to distinct flotation behaviors [12]. Analysis of spodumene crystals has indicated that oxygen atoms are the most active sites, readily combining with H^+^ in water, whereas lithium atoms exhibit low activity, requiring activators during flotation assessment. Examining the atomic bonding that takes place within the crystal suggests that mineral dissociation likely occurs along the weakest Li-O bond. Differences in the electronic structure are a key factor in flotation separation [13]. First-principle results show that the stronger covalence of the Fe-S bond in chalcopyrite enhances its hydrophobicity [14]. Studies of heteropolar ore have revealed strong Si-O bonds but very weak Zn-O bonds within crystal structures, causing preferential fractures along the Zn-O bonds during crushing [15]. This results in strong interactions with polar water molecules and poor natural floatability. Studying crystal electronic structures and bond populations can help predict minerals’ primary exposed cleavage surfaces [16]. Frontier orbital calculations indicate that N-(carboxymethyl)-N-tetradecylglycine exhibits better activity and stronger interaction with fluorapatite than oleic acid [17]. Similarly, tert-dodecyl mercaptan interacts more strongly with sphalerite than butyl xanthate [18], and frontier orbital theory has demonstrated that dodecylamine is a more effective collector for sphalerite than sodium oleate [19].

Research on fluorite’s crystal structure and frontier orbitals indicates that impurity addition enhances its reactivity with oleic acid [20]. Studies by Zhang et al. revealed significant differences in the metal atom activity between fluorapatite and dolomite crystals [21]. The O–Ca bond in fluorapatite exhibits a stronger ionic character, and the activity of Ca sites on its surface exceeds that found on dolomite. Comparative studies of fluorite and dolomite crystal properties remain scarce. The separation of fluorite from dolomite presents a critical challenge in fluorite ore flotation, making the investigation of both minerals’ crystal properties essential.

This study employed DFT calculations to optimize the structures of fluorite and dolomite. By analyzing the atomic coordination structures, DOS, Mulliken populations, and bond dissociation characteristics, we systematically compared the properties of their mineral crystals and surfaces. This approach deepened our understanding of the differences in crystal properties between fluorite and dolomite, providing theoretical guidance for flotation practices.

## 2. Results and Discussion

### 2.1. Crystal Structure Optimization

The crystal structure models of the fluorite and dolomite were sourced from the American Mineralogist Crystal Structure Database (AMCSD) [22,23]. Fluorite adopts a cubic crystal system with a space group of Fm−3m. Its unit cell parameters were as follows: a = b = c = 0.546 nm, α = β = γ = 90°, and Z = 4. Within the structure, the Ca^2+^ ions formed a cubic close-packed arrangement, with tetrahedral interstices being fully occupied by F^−^ ions. Each Ca^2+^ coordinate came with eight surrounding F^−^ ions, forming Ca–F8 cubic polyhedra, while each F^−^ was tetrahedrally coordinated by four Ca^2+^ ions, resulting in a regular tetrahedral arrangement.

Dolomite crystallizes in the trigonal system with a space group of R−3C. Its unit cell parameters, exhibiting pronounced trigonal symmetry, are as follows: a = b = 4.8498 Å, c = 15.9783 Å, α = β = 90°, γ = 120°, and Z = 3. It was found that the Ca^2+^ and Mg^2+^ cations occupied distinct lattice sites in an ordered, alternating manner. Planar triangular CO_3_^2−^ groups coordinated with the metal ions, forming layered structural units. Both Ca^2+^ and Mg^2+^ exhibited octahedral coordination, each bonded to six O^2−^ ions.

**Figure 1:**
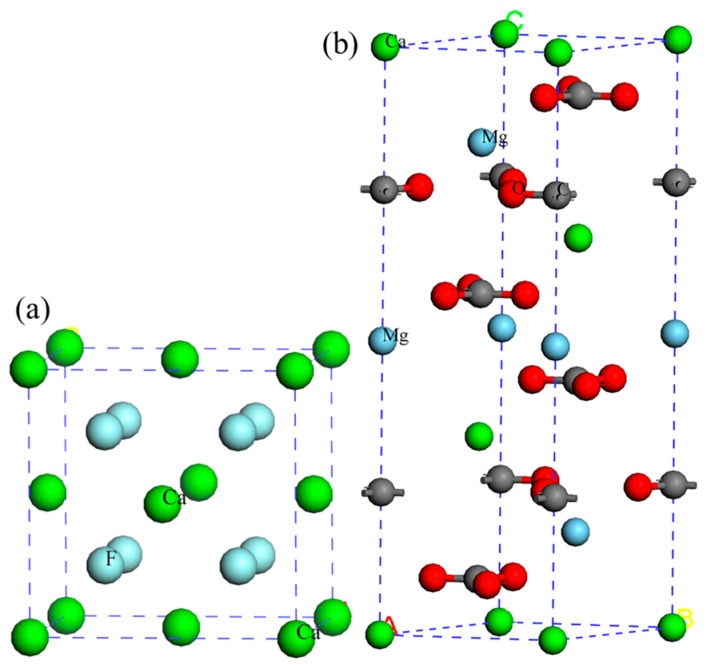
The crystal structures of fluorite (**a**) and dolomite (**b**).

The optimized crystal structures are depicted in Figure 1. The optimized unit cell parameters are summarized in Table 1. As evident from the table, the theoretically calculated lattice parameters (a, b, and c) for both fluorite and dolomite were found to be in excellent agreement with the experimental values with all deviations less than 1%. This close correspondence between theoretical calculations and experimental data indicates that the computational methodology employed was reliable.

### 2.2. Mulliken Population of Fluorite and Dolomite

Initial valence electron configurations for fluorite constituents prior to structural optimization were set as Ca (3s^2^ 3p^6^ 4s^2^) and F (2 s^2^ 2p^5^). Post-optimization Mulliken population analysis (Table 2) revealed the Ca valence configuration as 4s^0^·^15^ 3p^6^ 3s^2^ 4d^0.46^, with a total of 8.62 electrons localized on Ca. This corresponded to a net charge of +1.38e, indicating Ca acts as an electron donor. Charge depletion occurred primarily from the 4d orbital (−1.38e relative to the initial neutral configuration). Conversely, the optimized F configuration was 2s^1.96^ 2p^5.73^, localizing 7.69 electrons and acquiring a net charge of −0.69e, which is consistent with its role as an electron acceptor. Electron gain occurred predominantly in the 2p orbital (+0.73e).

Initial valence configurations for dolomite constituents were set as Ca (3s^2^ 3p^6^ 4s^2^), Mg (3s^2^), C (2s^2^ 2p^2^), and O (2s^2^ 2p^4^). Post-optimization Mulliken populations (Table 2) show the Ca atom with a net charge of +1.47e, indicating a loss of 1.47 electrons. Notably, 0.46 electrons occupied the Ca 3d orbital. The optimized C configuration was 2s^0.87^ 2p^2.39^. The increased 2p occupancy (relative to initial 2p^2^) likely arose from the electron transition from the depleted 2s orbital. Similarly, the Mg valence configuration was 3s^2.27^ 2p^6.40^ (implying significant p-orbital occupancy). Each O atom exhibited a configuration of 2s^1.80^ 2p^4.92^, corresponding to a net electron gain of 0.71e.

Bader charge analysis is a computational method that quantitatively investigates charge transfer phenomena by partitioning atomic volumes, and it is based on quantum mechanical electron density distribution [24]. Relevant studies demonstrate that, in fluorite, the Bader charge transfer amounts for Ca and F atom are approximately +1.4e and −0.7e, respectively, confirming its predominantly ionic bonding character [25]. For dolomite, the Bader charge transfer values are approximately +1.8e for Ca atoms and +1.2e for Mg atoms, revealing a mixed ionic–covalent bonding feature [26]. Additionally, intra- CO_3_^2−^ charge redistribution was observed: the carbon atoms exhibited a charge transfer of ~+0.4e, while the oxygen atoms showed ~−0.4e, reflecting the π-electron delocalization effect within the CO_3_^2−^ anion. These results are consistent with those obtained from Mulliken atomic population analysis.

As presented in Table 3, the Ca-F bond exhibited a Mulliken population of 0.09 with a bond length of 2.422 Å. This low population value signifies predominantly ionic character, which is consistent with the electron transfer from the electropositive Ca atom to the electronegative F atom. This ionic bonding arose primarily from the high propensity of Ca to lose its 4s electrons and the unsaturated nature of the F 2p orbitals, which readily accept electrons. The F-F interactions displayed negative population values, indicating the presence of an antibonding character. Given the F-F distance exceeding 2.5 Å, no significant chemical bonding exists between F atoms.

The Mulliken population data for dolomite bonds (Table 3) revealed Ca-O and Mg-O bond populations of 0.09 and 0.18, respectively. These values confirm the ionic nature of both bonds. The lower population for the Ca-O bond (0.09) compared to Mg-O (0.18) indicates weaker bonding interaction and, consequently, greater susceptibility to cleavage. In contrast, the C-O bonds demonstrate a significantly higher population of 0.86, approaching unity. This population value is characteristic of covalent bonding, reflecting high bond strength. Negative population values were observed for the O-O interactions, signifying an antibonding character. Based on bond populations and calculated bond lengths within the dolomite structure, the bond strength hierarchy was established as follows: C-O > Mg-O > O-O > Ca-O. The relative weakness of the Ca-O, (non-bonding) O-O, and Mg-O bonds facilitates their cleavage at crystal surfaces, leading to the formation of unsaturated bonds involving Ca, O, and Mg atoms.

### 2.3. Electron Density of Fluorite and Dolomite

**Figure 2:**
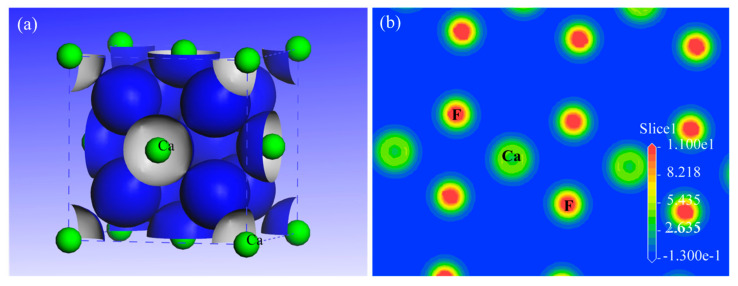
The charge density isosurface of the fluorite crystal (**a**), and the charge density on the {111} plane (**b**).

Analysis of the charge density isosurface (Figure 2a) revealed predominant electron localization between the Ca and F atomic sites in the fluorite crystal. The line profile of the charge density along the Ca-F bonding axis (Figure 2b) exhibited minimal overlap at the bond midpoint. This absence of covalent electronic sharing, characteristic of ionic bonding, confirms the exclusively ionic nature of the Ca-F interaction.

**Figure 3:**
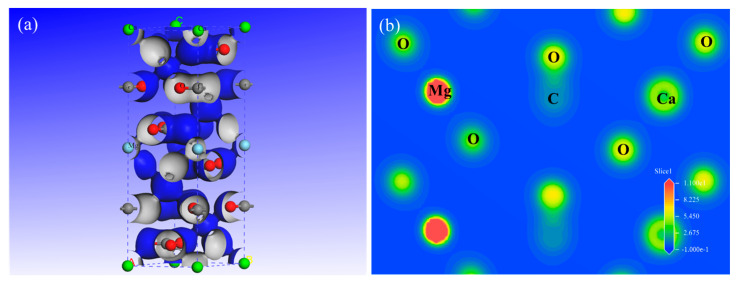
The charge density isosurface of the dolomite crystal (**a**), and the charge density on the {104} plane (**b**).

The charge density isosurface analysis (Figure 3a) demonstrated that the electron distribution in dolomite is primarily concentrated within the CO_3_ groups, with secondary accumulation observed in the Ca-O and Mg-O interatomic regions. Charge density profiles along the Ca-O and Mg-O bonding axes (Figure 3b) revealed minimal orbital overlap at bond midpoints, providing strong evidence for ionic interactions between these cation–anion pairs. In marked contrast, significant electron density overlap was observed between the C and O atoms within the CO_3_ groups (Figure 3b), which clearly manifested the covalent bonding nature of these carbonate units.

**Figure 4:**
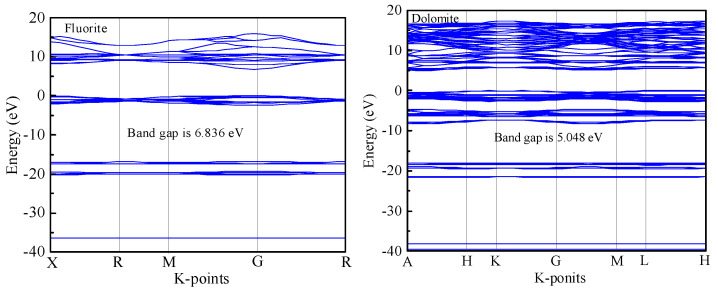
The band structures of the fluorite and dolomite crystals.

Figure 4 shows that the bandgap of fluorite was 6.836 eV, which is in good agreement with the calculated value of 6.902 eV reported in the literature [27]. The bandgap of dolomite was 5.048 eV, which is consistent with the calculated values of 5.07 eV [28], 5.035 eV [29], and 5.005 eV [30] from previous studies. While conductors possess an essentially negligible band gap (0 eV) and as semiconductors exhibit narrow gaps (typically < 2 eV), fluorite and dolomite demonstrate significantly wider band gaps that are characteristic of insulators [31]. These large band gaps preclude thermal excitation of electrons from the valence band to the conduction band at ambient conditions. Consequently, both materials exhibited negligible electrical conductivity due to the absence of mobile charge carriers in either band, classifying them as electrically insulating minerals.

### 2.4. State Densities of Fluorite and Dolomite

**Figure 5:**
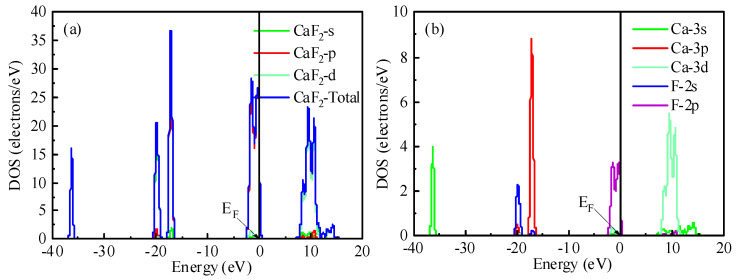
The total density of the state of fluorite (**a**), and the partial density of the state of Ca-F (**b**).

The total and partial density of the states (DOS/PDOS) for fluorite are presented in Figure 5. As shown in Figure 5a, the total DOS spanned an energy range from −40 eV to 20 eV. Detailed PDOS analysis (Figure 5b) revealed distinct orbital contributions: the Ca s orbital dominated the states between −37 eV and −35 eV, while strong hybridization occurred between the Ca p and F s orbitals in the region from −21 eV to −12 eV. Significant hybridization was also observed between the Ca (s, d) and F p orbitals within the energy windows of −3 eV to 0.5 eV and 5 eV to 15 eV. Crucially, near the Fermi level (E_F_), the valence band edge exhibited the foremost contribution from the F 2p orbital, with secondary contributions arising from the Ca 3s and Ca 4d states. Given that greater DOS intensities near E_F_ correlate with enhanced chemical reactivity for surface atoms, the dominant F 2p contribution suggests that fluorine atoms possess significant reactivity during chemical reactions.

**Figure 6:**
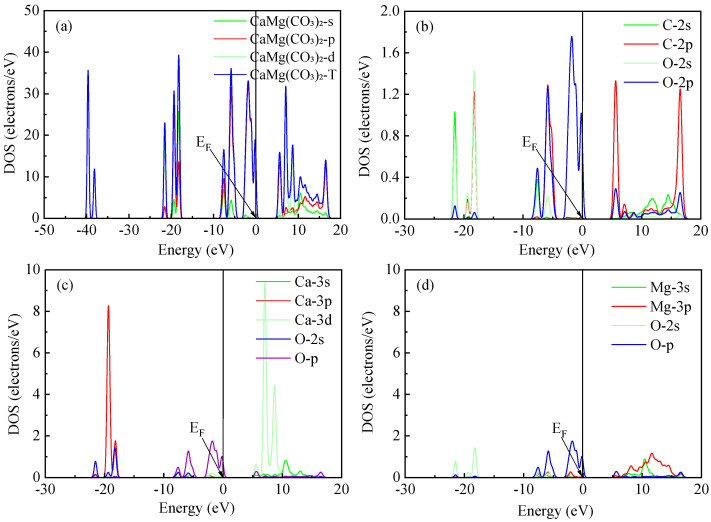
The total density of the state of dolomite (**a**). The partial density of the state of different atoms: C-O (**b**), Ca-O (**c**), and Mg-O (**d**).

The DOS and PDOS for dolomite are presented in Figure 6. Analysis of the total DOS (Figure 6a) showed it distributed across an energy range from −50 eV to 20 eV. Examination of the C-O PDOS (Figure 6b) revealed significant hybridization between the C 2p and O 2p orbitals within the energy window spanning −25 eV to 17 eV. This substantial orbital overlap is indicative of the covalent bonding character between carbon and oxygen atoms, and it is mediated primarily through their 2p orbitals. Conversely, the PDOS profiles for Ca-O (Figure 6c) and Mg-O (Figure 6d) exhibited distinct differences in both peak shapes and intensities for the Ca 3p and Mg 2p states, respectively. These contrasting features signify weak hybridization between the cation (Ca^2+^, Mg^2+^) and oxygen orbitals, which is characteristic of ionic bonding interactions. Notably, near the EF, the valence band edge was dominated by contributions from the O 2p orbital, with adjacent contributions originating from the Ca 3p states. Consequently, these findings suggest that oxygen and calcium atoms exhibit enhanced chemical reactivity within the dolomite structure relative to magnesium.

### 2.5. The Cleavage Characteristics of Fluorite and Dolomite

**Figure 7:**
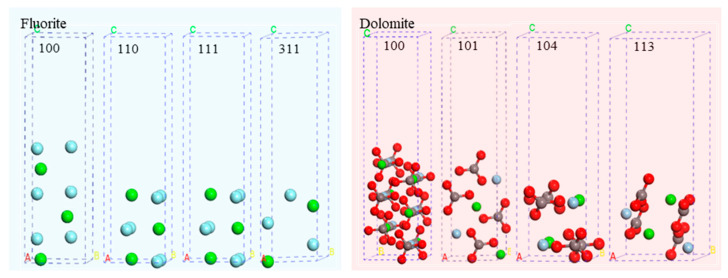
The structure of the various cleavage surfaces of fluorite and dolomite.

The fluorite XRD analysis identified prominent diffraction peaks corresponding to the {111}, {220}, {311}, and {400} cleavage planes, indicating their preferential exposures during mechanical fracture and grinding processes. Given that the cleavage planes {220}/{110} and {400}/{100} shared the same cleavage orientation and were parallel crystallographic planes, the surfaces under investigation included the {111}, {110}, {311}, and {100} planes. Figure 7 and Table 4 present the crystallographic orientations and their respective unsaturated bond densities. Unsaturated bond density refers to the number of unsaturated chemical bonds exposed per unit area of the fracture surface. A higher unsaturated bond density indicates more exposed unsaturated bonds on the fracture surface, resulting in stronger surface chemical activity. As quantified in Table 4, the unsaturated bond density (number per unit area) progressed in the following hierarchy: {311} > {100} > {110} > {111}. The {111} facet exhibited the minimal unsaturated bond density (14.76 nm^−1^) and the maximal interlayer spacing (0.4713 nm) among all the studied planes. Consequently, fluorite demonstrated the highest propensity for preferential cleavage along {111} when under external stress, and this was followed by the {110} and {100} orientations. Conversely, the {311} plane displayed pronounced cleavage resistance due to its elevated unsaturated bond density.

The dolomite XRD analysis identified distinct diffraction peaks associated with the {104}, {113}, {202}, and {100} cleavage planes, thus confirming their preferential exposure during mechanical comminution. Based on the parallelism between the {202} and {102} planes, the examined surfaces included {104}, {113}, {102}, and {100}. Table 4 data revealed the unsaturated bond density sequence: {104} > {101} > {113} > {100}11. The {104} plane possessed the lowest unsaturated bond density (10.55 nm^−1^) and largest interlayer spacing (0.3899 nm) within the evaluated facets. Thus, the dolomite underwent preferential cleavage along {104} when under mechanical stress, a conclusion corroborated by the XRD analysis.

## 3. Material and Methods

### 3.1. Materials

**Figure 8:**
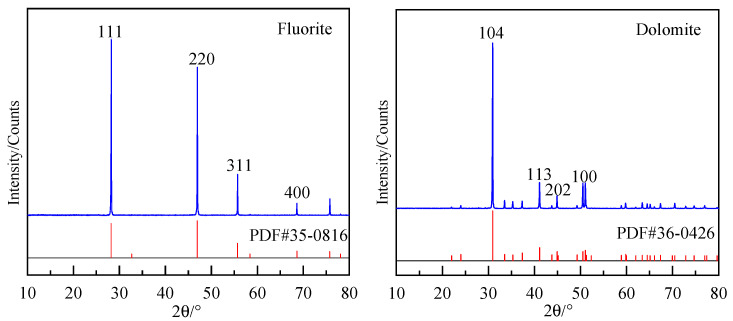
XRD diffractograms of fluorite and dolomite.

The fluorite samples sourced from Erenhot, Inner Mongolia, and the dolomite samples from Haicheng, Northeast China, were selected based on high purity. Monolithic specimens underwent comminution procedures (crushing, grinding, and sieving) to obtain sub-5 μm particles for XRD analysis. The mineral purity was verified by X-ray diffraction (XRD) analysis, as demonstrated in Figure 8.

### 3.2. First-Principle Calculations

The first-principle calculations of the crystal structure were completed via the CASTEP module from Material Studio software [32]. The PBE functional in the Generalized Gradient Approximation (GGA) was used to describe the exchange correlation potential [33]. The cut-off value of the plane wave energy was set to 450 eV. The tolerance for self-consistent calculation was set to 5.0 × 10^−5^ eV/atom. Maximum displacement values of less than 0.005 Å and maximum atomic force values of less than 0.1 eV/Å were used as the convergence thresholds for geometric optimization.

Formulas (1) and (2) were then used to calculate the fracture bond density and surface energy of the different crystal faces of fluorite and dolomite:

(1) $$D_{b}=\frac{N_{b}}{s},$$

(2) $$E_{surf}=\frac{E_{slab}-\left(\frac{N_{slab}}{N_{bulk}}\right)E_{bulk}}{2A}.$$

In the above formulas, *D_b_* and *N_b_* represent the cleavage plane’s broken bond density and the number of broken bonds per unit cleavage plane, respectively; *s* denotes the area of the cleavage plane (m^2^); *E_surf_* corresponds to the surface energy (J/m^2^); *E_slab_* is the total energy of the surface structure (J); *E_bulk_* signifies the total energy of the mineral unit cell (J); *N_bulk_* indicates the number of atoms in the optimized mineral unit cell; *N_slab_* refers to the number of atoms in the surface structure; and *A* is the area of the calculated surface (m^2^).

## 4. Conclusions

Fluorite and dolomite exhibit fundamentally distinct bonding architectures. Fluorite features purely ionic Ca-F bonds (bond length: 2.422 Å; Mulliken population: 0.08), whereas dolomite exhibits a layered ionic–covalent framework characterized by strong C-O covalent bonds (1.295 Å, 0.86 population) alongside weaker Ca-O/Mg-O ionic interactions. Electronic structure analysis confirms fluorite’s insulator properties (bandgap: 8.016 eV) and fluorine’s surface reactivity (domination of F 2p density of states), while dolomite’s reactivity stems from the oxygen/calcium states near the Fermi level. These differences underlie their distinct interfacial behaviors during flotation.

The {111} cleavage plane of fluorite (lowest unsaturated bond density: 14.76 nm^−1^) and the {104} plane of dolomite (10.55 nm^−1^) dictate their mechanical fracture patterns. Fluorite’s ionic lattice preferentially interacts with anionic collectors, whereas dolomite’s mixed bonding necessitates tailored reagent design. This work establishes a foundation for developing selective separation strategies by exploiting crystal-specific surface properties.

For cationic flotation reagent design, compounds demonstrating strong fluorine affinity, but relatively weaker oxygen binding capacity, should be prioritized to enhance separation selectivity. When employing anionic reagents, the electronic property differences between surface active sites (calcium ions) should be leveraged to select polar groups exhibiting optimal compatibility with fluorite’s electronic characteristics, thereby improving selective separation performance.

## Figures and Tables

**Table 1:** The lattice parameters of fluorite and dolomite.

Lattice Parameter	Theoretical Calculation Value (Å)		Experimental Value (Å)	
	Fluorite	Dolomite	Fluorite	Dolomite
a	5.593616	4.862532	5.56784	4.8069
b	5.593616	4.862532	5.56784	4.8069
c	5.593616	16.234903	5.56784	16.003

Å: Ångström, used to measure small distances at the atomic and molecular levels.

**Table 2:** The Mulliken atomic populations of fluorite and dolomite.

Minerals	Species	s	p	d	Total/e	Charge/e
Fluorite	Ca	2.15	6.00	0.46	8.62	1.38
	F	1.96	5.73	-	7.69	−0.69
Dolomite	Ca	2.10	6.00	0.44	8.53	1.47
	Mg	2.27	6.40	-	8.66	1.34
	C	0.87	2.39	-	3.26	0.74
	O	1.80	4.92	-	6.71	−0.71

**Table 3:** The Mulliken bond populations of fluorite and dolomite.

Minerals	Bonds	Population	Bond Length/Å
Fluorite	Ca-F	0.08	2.422
	F-F	−0.03	2.797
Dolomite	Ca-O	0.09	2.408
	Mg-O	0.18	2.116
	C-O	0.86	1.295
	O-O	−0.22	2.243

Å: Ångström, used to measure small distances at the atomic and molecular levels.

**Table 4:** The broken bond densities of the fluorite and dolomite crystals with different surfaces.

Minerals	Surface	Formula of Unit Area, A (nm^2^)	Nb	Db (nm^−1^)	d (nm)
Fluorite	{100}	0.3955 × 0.3955 × sin90° = 0.1564	4	25.5754	0.1894
	{110}	0.5594 × 0.3955 × sin90° = 0.2212	4	18.0832	0.2681
	{111}	0.3955 × 0.3955 × sin60° = 0.1355	2	14.7601	0.4713
	{311}	0.6851 × 0.3955 × sin73.22° = 0.2594	12	46.2606	0.1548
Dolomite	{100}	0.4863 × 1.6235 × sin90° = 0.7895	16	20.27	0.1121
	{101}	0.6096 × 0.4863 × sin66.50° = 0.2719	6	22.07	0.0865
	{104}	0.7798 × 0.4863 × sin90° = 0.3792	4	10.55	0.3899
	{113}	0.8422 × 0.6096 × sin76.6888 = 0.4996	8	16.01	0.1838

## Data Availability

All data generated or analyzed during this study are included in this published article.
